# Complement C3 mediates podocyte injury through TLR4/NFΚB-P65 signaling during ischemia–reperfusion acute kidney injury and post-injury fibrosis

**DOI:** 10.1186/s40001-023-01054-1

**Published:** 2023-03-27

**Authors:** Yi Chen, Liyu Lin, Siyi Rao, Xuan Tao, Jiong Cui, Jianxin Wan

**Affiliations:** 1 Department of Nephrology, Blood Purification Research Center, The First Affiliated Hospital, Fujian Medical University, Fuzhou, 350005 China; 2 Fujian Clinical Research Center for Metabolic Chronic Kidney Disease, The First Affiliated Hospital, Fujian Medical University, Fuzhou, 350005 China; 3 Department of Nephrology, National Regional Medical Center, Binhai Campus of the First Affiliated Hospital, Fujian Medical University, Fuzhou, 350212 China; 4 Department of Pathology, The First Affiliated Hospital, Fujian Medical University, Fuzhou, 350005 China

**Keywords:** Acute kidney injury, Chronic kidney disease, Podocyte, Complement C3

## Abstract

**Background:**

The aim of this study was to explore the mechanism of complement C3a mediating podocyte injury during ischemia–reperfusion acute kidney injury (IR-AKI) and post-injury fibrosis.

**Methods:**

Renal artery clamping was used to establish IR-AKI and post-injury fibrosis model. HE and Masson staining were performed to observe renal fibrosis. The protein abundance levels were measured along with inflammatory markers, renal complement C3. Podocytes were treated with C3a with or without Toll-like receptor 4(TLR4) inhibitor. The effects of TLR4 up-regulation by TLR4 plasmids were examined.

**Results:**

C3^−/−^ resulted in amelioration of renal dysfunction by reducing podocyte damage and renal fibrosis. Immunoblot with renal tissue homogenates from IR-AKI mice revealed that C3^−/−^ decreased TLR4/Nuclear Factor-κB (NFκB)-P65.

**Conclusion:**

Our results indicate that modulating C3/TLR4/NFκB-P65 signaling pathway is a novel therapeutic target for the IR-AKI and post-injury fibrosis.

**Graphical Abstract:**

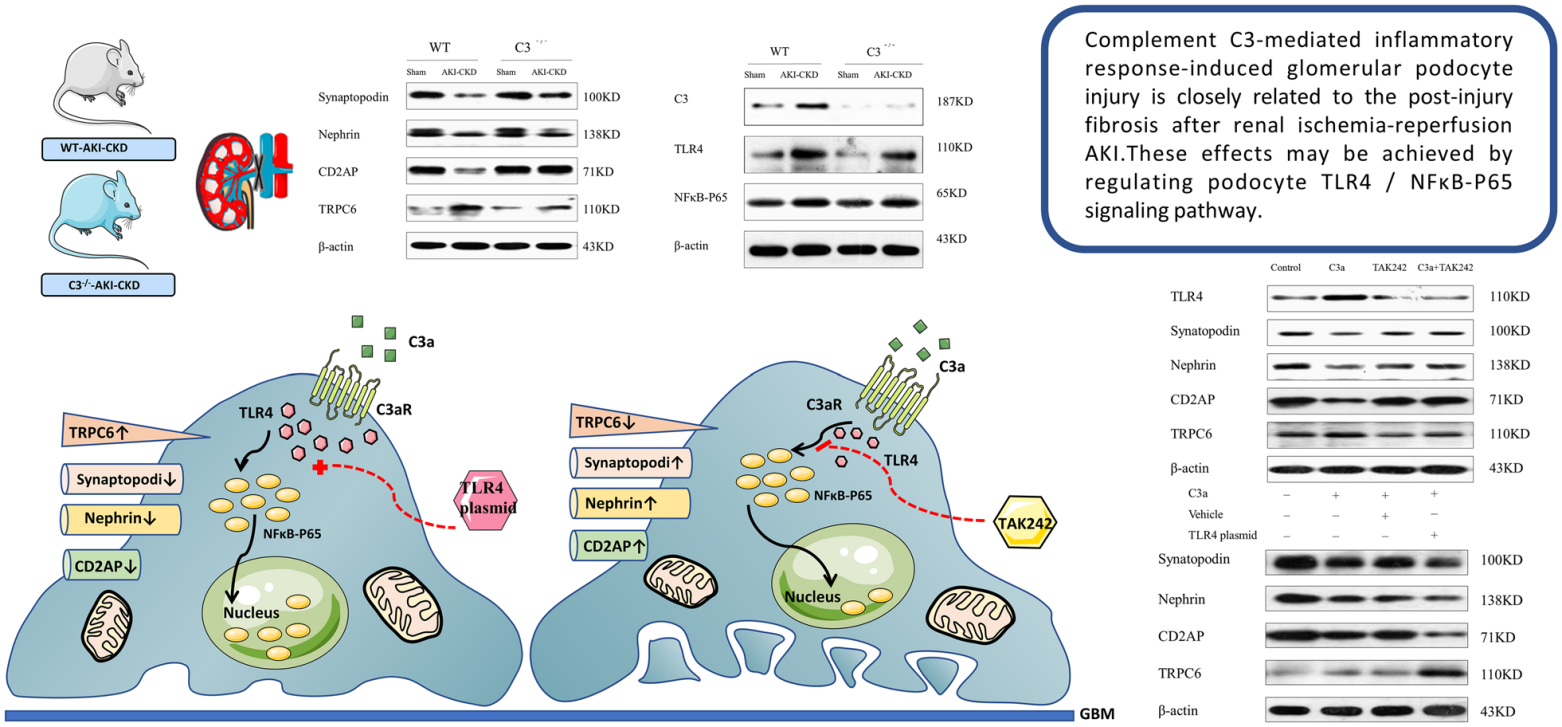

## Introduction

Chronic kidney disease (CKD) has increased significantly; there were over 697 million patients worldwide in 2019 [1]. The prevalence rate of CKD in Chinese adults is up to 10.8% [2]. The incidence rate of acute kidney injury (AKI) inpatients in Chinese general hospitals is 5–7%; approximately 50% of surviving patients suffer from permanent renal dysfunction, poor prognosis, and heavy medical burden [3–5]. As far back as 2009, the international nephrology community has clearly indicated that AKI is the direct cause of CKD [6]; however, for many years, the treatment of AKI has been symptomatic only, preventing AKI from progressing to CKD has become the focal point of the international nephrology community.

The clear pathophysiological mechanisms related to chronicity after AKI are microvascular endothelial cell damage, inflammation, and abnormal renal tubular epithelial cells activation. However, there have been few studies on podocytes. Perspicacious studies found that patients with AKI often display persistent proteinuria and albuminuria after AKI, which is closely related to subsequent CKD [7]. Podocyte damage leads to proteinuria, which is an indicator of most glomerular diseases and is related to the progression of kidney disease. Research by Hu et al. suggested that podocytes involve in the occurrence and progression of AKI [8].

The role of complement in IRI has received increasing attention. However, the mechanism is not clear. Complement deficiency can inhibit elevated TLR4 [9]. Inhibition of TLR4 attenuated Heme-induced complement deposit on endothelial cells. A central role of P-sel is to tag endothelium as a target for complement activation in vivo and provide the missing link between TLR4 and complement activation [10]. Recent research found that less secretion of C3 appears to inhibit the activation of the High-Mobility Group Box 1 (HMGB1)-TLR4-p65 pathway signal pathway and the production of transforming growth factor-β (TGF-β1), thereby alleviating renal fibrosis in unilateral ureteral obstruction (UUO) mice [11]. However, whether C3 and TLR4 interact in CKD after AKI is still unclear.

In previous research, we established unilateral renal ischemia IR-AKI model under different ischemia times and found that the mice in the 20 min ischemia group were mainly characterized by mild AKI lesions, without chronic renal manifestation after AKI. Mice in 40 min ischemia group showed severe renal tubule and renal interstitial damage, which was irreversible. However, ischemia 30 min and contralateral kidney excision after 8 days could establish a post-AKI fibrosis kidney model. Electron microscopy was confirmed in mouse podocytes during AKI injury, proteinuria, and subsequent chronic kidney fibrosis, thus confirming that podocyte injury is an important cause of AKI and post-AKI renal fibrosis [12]. Our research found that complement C3 exacerbates renal interstitial fibrosis by facilitating the M1 macrophage polarization in a mouse model of unilateral ureteral obstruction [13]. Complement C3 activation also generates the anaphylatoxins C3a, which have potent proinflammatory effects. Therefore, we hypothesize that complement C3-mediated podocyte injury is involved in the post-injury fibrosis of the kidney after AKI. This study used wild-type C57BL/6 mice and C3 knockout mice to establish an IR-AKI and post-injury fibrosis model. Additionally, by in vitro treatment of mouse podocytes cultured under ischemic and hypoxic conditions, the mechanism of complement C3 promoting IR-AKI and post-injury fibrosis was investigated through examination of the TLR4/NFκB-P65 signaling pathway. Our findings aim to provide new outlooks on the prevention and treatment of post-AKI kidney fibrosis.

## Materials and methods

### Animals care

All mice were raised in specific pathogen-free (SPF) barrier facility at The Laboratory Animal Center of Fujian Medical University. All procedures were approved by The Animal Welfare and Ethics Committee of Fujian Medical University (Approval Number: 2017-062): Ninety 16-week-old C57BL/6 mice (weight: 25–28 g, Shanghai SLAC Laboratory Animal Co., Ltd (production license number: SCXK (Shanghai) 2012-0002)) and twenty C3^−/−^ mice (C3-deficient mice (strain B6.129S4-C3tm1Crr) of C57BL/6 genetic background from Jackson Laboratory (Bar Harbor, ME), 5 mice per cage (365 mm × 207 mm × 140 mm; model GK, Suzhou Feng's Laboratory Animal Equipment Co. Ltd, China) with bedding material (cellulose pellet) housed at a room temperature of 22 ± 2 ℃, the humidity of 55 ± 5%, and 12-h light/dark cycle. Mice had unrestricted get food and water.

### Establishment of mouse ischemia–reperfusion acute kidney injury and post-injury fibrosis model.

Establishment of mouse IR-AKI and post-injury fibrosis model was according to Yi Chen method [12]. Fifty C57BL/6 mice were intraperitoneally anesthetized with 3% pentobarbital sodium (1.0–1.5 mL/kg). The left renal artery was clamped with a micro-arterial clip for 30 min to restore blood flow. Sham group mice underwent the same surgical procedure without clamping the left renal artery. One week after surgery, the right kidney was excised. Afterward, the mice (*n* = 10) were euthanized at 28 days, blood and kidney tissues were also obtained for subsequent analyses. Euthanasia is performed as follows: pentobarbital sodium (60 mg/kg) is injected intraperitoneally and the animal is executed by cervical dislocation after 10 min. C3^−/−^ (C57BL/6 background) mice AKI model were received the same method.

### Determination of urine protein and renal function

Urinary protein, serum creatinine (Scr), and urea nitrogen (BUN) were examined with biochemical analyzer.

### Hematoxylin–Eosin (HE) and Masson’s staining

The left kidney was excised, 10% formalin fixed, paraffin embedded, and 3 μm serial sectioned. Subsequently, sections were dewaxed, hydrated, hematoxylin–eosin (HE) stained, and Masson stained.

### Renal tissue pathological damage score:

Mouse kidney tissue specimens were stained by HE staining and Masson’s staining, and renal pathology scores were evaluated with light microscope as described previously [12]. For each animal, 10 randomly selected nonoverlapping interstitial fields and glomerulus were analyzed, and their average was used as data from one animal sample. And the average value was taken for statistical analysis. An independent pathologist was responsible for pathological scoring according to the following criteria:

- Glomerular mesangial hyperplasia: no, mild, moderate, and severe mesangial hyperplasia were scoring as 0, 1, 2, and 3 points, respectively.
- Degree of glomerulosclerosis: glomerulosclerosis rate ≤ 0%, < 25%, 25% − 50%, and > 50%, were scoring as 0, 1, 2, and 3 points, respectively.
- Renal tubular interstitial score (RTIS), namely, (1) renal tubular degeneration and necrosis, (2) renal tubular atrophy, (3) interstitial inflammatory cell infiltration, and (4) interstitial fibrosis. According to the extent and severity of the lesions, 0, 1, 2, and 3 were scored as none, < 25%, 25−50%, and > 50%, respectively.

### Evaluation of the glomerular and podocyte morphological changes by transmission electron microscopy (TEM)

Kidney cortex tissue was obtained, cut out into a 1 mm3 tissue block, and placed in an electron microscope fixative solution. The tissue was then alcohol-acetone dehydrated, embedded, and sliced sections were observed for podocyte ultrastructure and glomerulosclerosis by electron microscope.

### Immunohistochemistry

Paraffin sections were sliced and baked, dewaxed and hydrated, and incubated in 3% (hydrogen peroxide solution) H_2_O_2_ for 10 min. Sections were incubated in primary antibody (50 μL/piece). Primary antibodies concentrations were Nephrin (Cat.ab2163, Abcam, 1:200), CD2AP (Cat.5478, CST, 1:100), Synaptopodin (Cat.ab22449, Abcam, 1:200), transient receptor potential channel 6 (TRPC6) (Cat.ab233413, Abcam, 1:100), C3 (Cat.ab200999, Abcam, 1:200), TLR4 (Cat.ab13867, Abcam, 1:200), and NFκB-P65 (Cat.3033, CST, 1:200), at 37℃ for 1 h, and rinse with phosphate-buffered saline (PBS). Then they were incubated in secondary antibody (China Zhongshan Golden Bridge) at 37 ℃ for 30 min, followed by rinse with PBS for 10 min × 3. Then after 1–2-min DAB incubation and sections were subsequently hematoxylin counterstained, dehydrated, and sealed. PBS replaced the primary antibody in all negative controls. Sections were evaluated under the microscope at a field of view of 200 times magnification. Five fields were randomly selected from each slice and optical density (IOD) value analysis was performed with the image analysis system, Motic Images Advanced. The protein expression level was represented by IOD value.

### Western blot analysis

Kidney cortex tissues were lysate with RIPA lysate. 1 × sodium dodecyl sulfate (SDS) lysis buffer was used to extract total proteins of glomerular podocytes. The protein content was determined according to the instructions of a Braford protein quantification kit. Protein extract was separated with 10% SDS-PAGE, transferred to polyvinylidene fluoride (PVDF) membrane, and incubated in 5% free-fat milk at room temperature for 30 min. The concentrations of antibodies were Nephrin (Cat.ab2163, Abcam, 1:400), CD2AP (Cat.5478, CST, 1:1000), Synaptopodin (Cat.ab22449, Abcam, 1:2000), TRPC6 (Cat.ab233413, Abcam, 1:1000), TLR4 (Cat.ab13867, Abcam, 1:1000), NFκB-P65 (Cat.3033, CST, 1:1000), and incubation at 37℃ for 1.5 h. The membrane was rinsed with PBS for 10 min × 3, incubated in secondary antibody (1: 2000) at 37 ℃ for 1 h, rinsed with PBS for 10 min × 3, exposed, developed, and analyzed with White/Ultraviolet Transilluminator system software. The target protein/β-actin (optical density, OD) ratio was used as the indicator of the target protein level.

### Podocyte culture

HSMPs (heat-sensitive mouse podocytes) were donated by Professor Ding Guohua of the School of Medicine of Wuhan University. HSMPs culture protocol has been previously published [14]. In vitro HSMPs were propagated under undifferentiated conditions (33 ℃, 5% CO2, 1640 medium containing 100 U/mL interferon (IFN-γ Sigma, USA) and 10% Fetal Bovine Serum, FBS) for 3–4 days. HSMPs were transferred to differentiation conditions (37 ℃, 5% CO2, 1640 culture medium without interferon, and 10% FBS). HSMPs morphology was observed under a microscope after 14 days. Differentiated podocytes were used experiment. For interruption of the TLR4, podocytes were treated with TAK242 (5 μM), a small-molecule-specific inhibitor of TLR4 signaling for 12 h.

### Podocyte culture ischemia and hypoxia conditioning

Differentiated podocytes were seeded on a 24-well culture plate. When the cells grew to 70–80% confluence, they were replaced in serum-free 1640 medium (1 mL/well) and incubated for 24 h [15]. Buffer liquid in ischemia and hypoxia groups were replaced with NaHCO3 4.5 mM, Na2HPO4 0.8 mM, NaH2PO4 0.2 mM, NaCl 106 mM, KCl 5.4 mM, CaCl2 1.2 mM, MgCl2 0.8 mM, MES 20 Mm, sugar free, and PH 6.6 and placed in a three-gas incubator (0.5% O2 + 5% CO2 + 94.5% N2, 37 ℃) for 24 h.

### Podocyte nuclear protein extraction

Podocytes were harvested with trypsin-Ethylenediaminetetraacetic acid (EDTA) and centrifuged at 500 × g for 5 min. Nuclear and cytoplasmic extracts were performed according to the NE-PER Nuclear and Cytoplasmic Extraction Reagents (Thermo Corporation) protocol. Extracts were stored at − 80℃ until use.

### F-actin staining of phalloidin podocyte cytoskeleton:

Cells were mounted on a slide, rinsed 3 times with PBS, and fixed with 4% paraformaldehyde for 15 min, followed by rinse with PBS for 3 min × 3. Phalloidin staining solution was incubated at 37 ℃ for 1 h and rinsed with PBS for 3 min × 3. DAPI was incubated for 15 min. The specimens were stained and washed with PBS for 5 min × 4. The cytoskeletal characteristics of podocytes were observed under a Confocal Laser Scanning Microscope and images were collected.

### Transfection of podocytes with the TLR4 plasmid vector

The transfection of TLR4 plasmid was executed according to the instructions of the liposome transfection kit (Liposome transfection kit X-treme GENE Transfection Solution (Invitrogen)). Upon completion, a new serum-free medium was replaced for the intervention test 24 h later.

### Statistical processing

All data are shown as mean ± Standard Deviation (SD) ($\overline{x}$± S). One-way ANOVA was performed for comparison between multiple groups, and LSD test for pairwise comparison between multiple groups; *P* < 0.05 indicated statistical significance.

## Results

### Changes in C3 and renal function during AKI

**Fig. 1:**
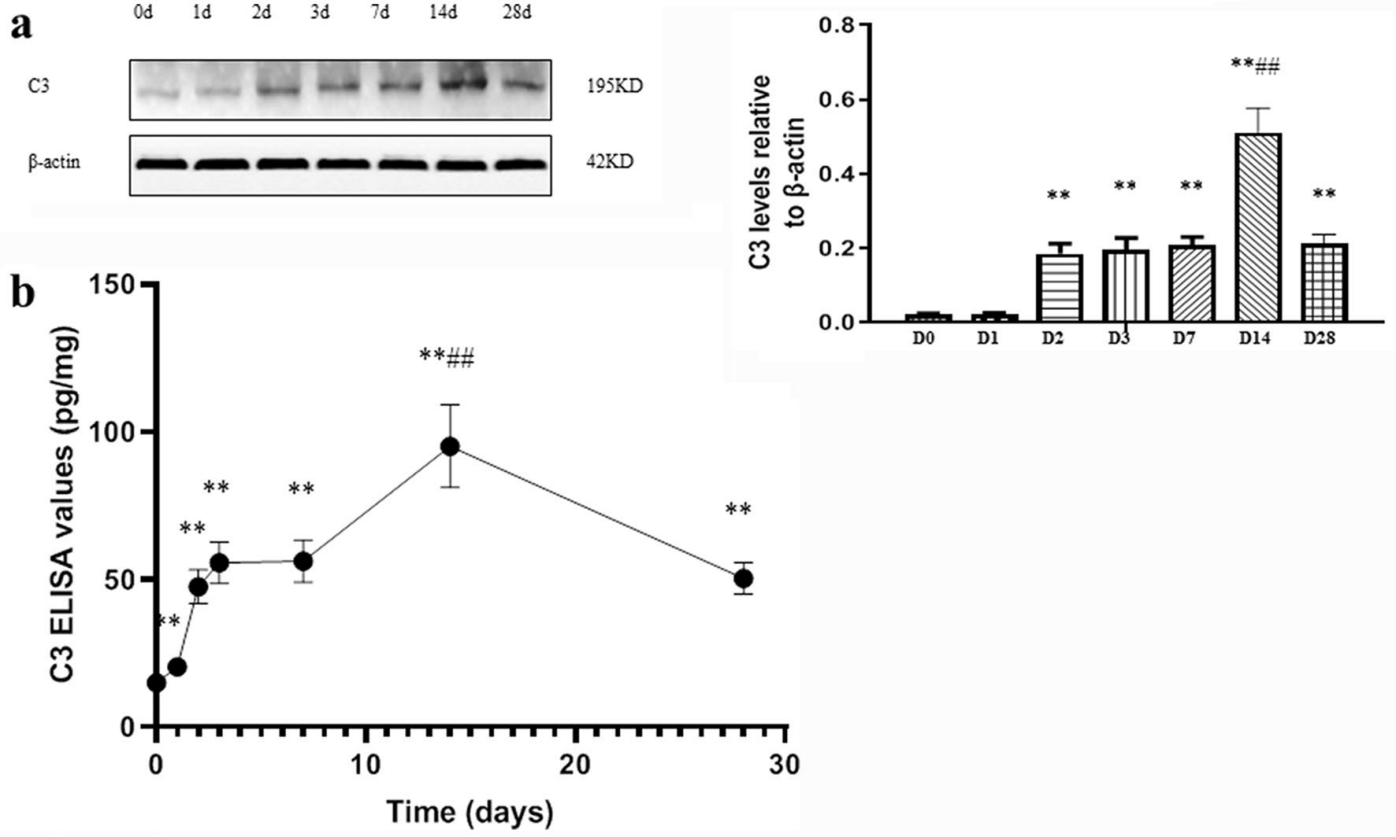
Changes in C3 during post-injury fibrosis. **a** Western blot gel electrophoresis film exposure image and quantitative analysis of the result, expressed as the ratio of target protein/β-actin (OD). **b** The levels of complement C3 in renal tissue were measured by ELISA method, n = 10. ** *P* < 0.01vs 0d; ##*P* < 0.01 vs 7d

To study the role of the **C3** in the development of renal in AKI, we detected the level of C3, urinary protein excretion (UPE), BUN, and Scr in ischemia–reperfusion injury (IRI) mice. Compared with Day 0, the expression of C3 increased at the early stage of renal injury. On Day 2, the level of C3 increased (*P* < 0.01), peaking on Day 14 and then slowly declined (*P* < 0.01) (Fig. 1).

Compared with the sham group, serum Scr and BUN levels in the AKI-CKD group on Day 1 were higher (*P* < 0.01). Compared with Day 1, the Scr and BUN levels of Day 2 were decreased (*P* < 0.01). Scr and BUN increased consistently through the period between Day 3 and Day 28 (*P* < 0.01). Compared with the sham group, the urine protein level in the AKI-CKD group was increased consistently through the period between Day 3 (*P* < 0.01) and Day 28 (*P* < 0.01) (Table 1).

**Table 1:** Changes in urinary protein, BUN, and Scr in each group ($\overline{x}$±s, n = 10)

	Group	D0	D1	D2	D3	D7	D14	D28
UPE (mg/d)	Sham	1.39 ± 0.19	1.43 ± 0.17	1.45 ± 0.18	1.44 ± 0.19	1.432 ± 0.16	1.41 ± 0.17	1.52 ± 0.27
	AKI-CKD	1.41 ± 0.15	1.91 ± 0.33	1.96 ± 0.27	2.22 ± 0.35**##	3.81 ± 1.23**##	12.05 ± 2.38**##	17.79 ± 2.86**##
BUN(mmol/L)	Sham	11.21 ± 1.25	11.01 ± 1.03	11.88 ± 0.69	11.92 ± 1.27	12.51 ± 1.42	12.15 ± 1.41	11.97 ± 1.55
	AKI-CKD	11.34 ± 1.37	29.97 ± 2.12**##	16.53 ± 1.24**##	22.79 ± 313**##	25.45 ± 2.99**##	29.68 ± 1.45**##	48.06 ± 6.72**##
Scr(umol/L)	Sham	7.08 ± 0.82	7.15 ± 0.72	7.19 ± 0.91	7.07 ± 0.81	7.12 ± 0.83	6.98 ± 0.69	7.25 ± 0.94
	AKI-CKD	7.43 ± 0.73	18.11 ± 1.68**##	11.75 ± 1.27**##	13.97 ± 1.79**##	15.68 ± 1.35**##	24.01 ± 1.29**##	35.61 ± 3.08**##

*UPE* urinary protein excretion, *BUN* serum urea nitrogen, *Scr* serum creatinine

^*^P < 0.05 vs Sham, **P < 0.01 vs Sham, #*P* < 0.05 vs D0, ##*P* < 0.01vs D0

### The effect of C3 on renal function and renal pathology during post-injury fibrosis

**Fig. 2:**
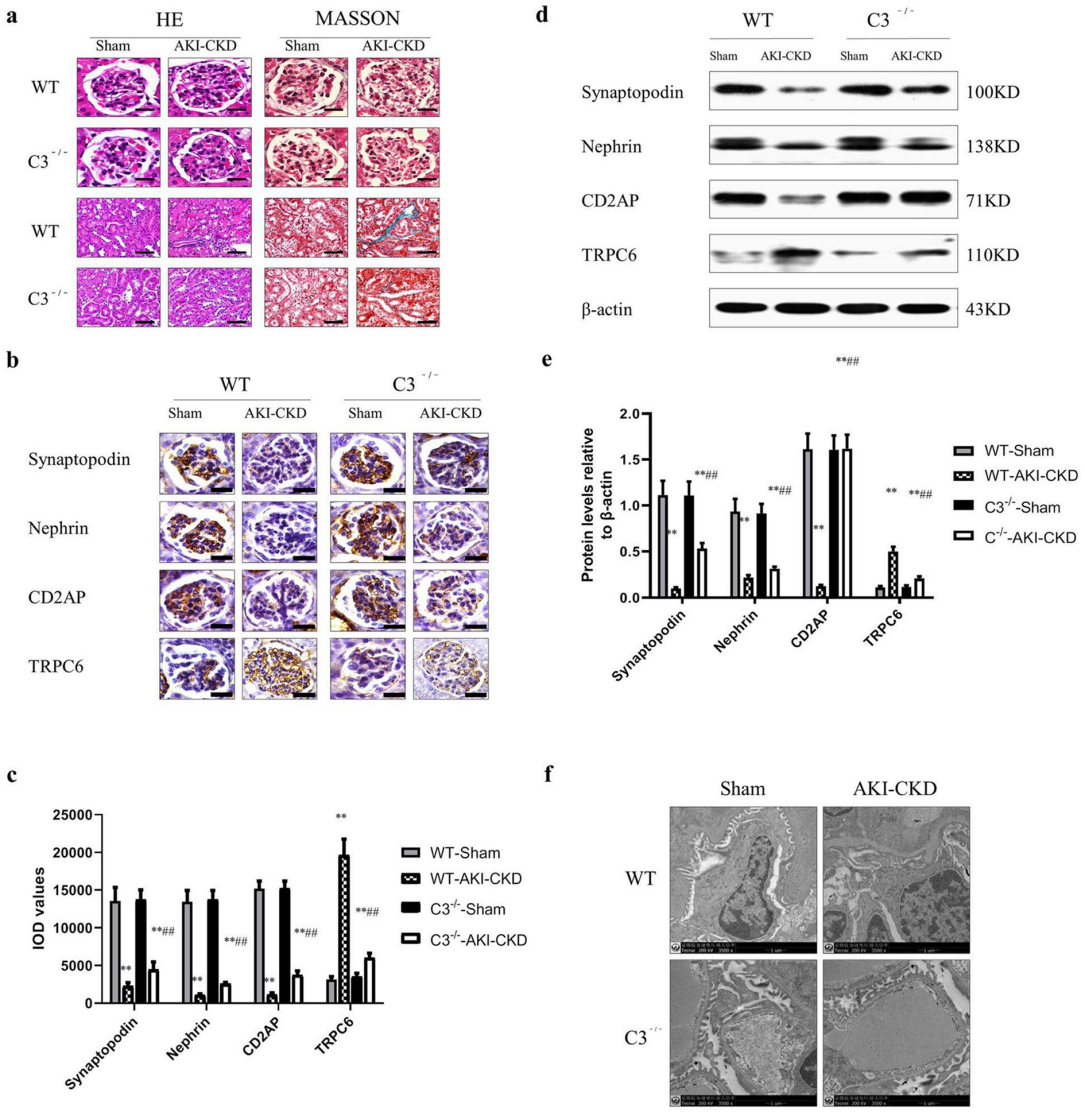
The effect of complement C3 on renal pathology during post-injury fibrosis. **a** Hematoxylin and eosin (HE) stain and Masson’s trichrome of kidney tissue. Scale bar of glomerular = 25 μm; Scale bar of renal tubular = 50 μm. **b** Immunohistochemical detection of the expression of Synaptopodin, Nephrin, CD2AP, TRPC6 (Scale bar = 25 μm) in mice of each group. **c** Compare the levels of podocyte functional protein (IOD) of kidney tissues in each group, n = 10. **d** Western blot gel electrophoresis film exposure image **e** Quantitative analysis of the result, expressed as the ratio of target protein/β-actin (OD), n = 10. **f** Morphological changes of glomerular podocytes in each group of mice (TEM, × 3500): kidney cortical tissue was cut to 1mm3 tissue for electron microscopy, n = 10. ** *P* < 0.01vs WT-sham or C3^−/−^-sham, ##* P* < 0.01vs WT-AKI-CKD

To determine whether C3 mediates renal fibrosis after IRI, we assessed renal pathology in C3^−/−^ mice compared with age-matched wild-type (WT) mice. Compared with the WT-sham group, the UPE, BUN, and Scr levels of the WT- AKI-CKD group and the C3^−/−^-AKI-CKD group were higher (*P* < 0.01). Compared with the WT -AKI- CKD group, the UPE, BUN, and Scr levels of the C3^−/−^-AKI-CKD group were statistically lower (*P* < 0.01) (Table 2). HE staining showed that the glomerular and renal tubular structures were intact and clear in the WT-sham group and C3^−/−^-sham group. In the WT-AKI-CKD group, mesangial cells hyperplasia and interstitial inflammatory cells were observed. Renal tubules were atrophy and interstitial structures were blurred. Meanwhile, in the C3^−/−^-AKI-CKD group, only a small amount of mesangial cells hyperplasia and fewer interstitial inflammatory cells were seen. Tubular and interstitial structures were relatively intact (Fig. 2a). Compared with the Sham group, the AKI-CKD group renal tissue pathological damage grade and score were raised (*P* < 0.01). And compared with the WT-AKI-CKD group, the C3^−/−^-AKI-CKD group had a lower grade and score of renal histopathological damage. However, there was no statistical difference between the WT-sham group and the C3^−/−^-sham group (*P* > 0.05) (Table 2).

**Table 2:** Comparison of renal pathological changes and renal function damage of mice in each group ($\overline{x}$ ± SD, n = 10)

	WT		C3^−/−^	
	Sham	AKI-CKD	Sham	AKI-CKD
Mesangial hyperplasia score	0 ± 0	1.83 ± 0.45^**^	0 ± 0	1.15 ± 0.25^**#^
Glomerulosclerosis score	0.11 ± 0.28	1.36 ± 0.33^**^	0 ± 0	0.76 ± 0.08^**#^
Tubular interstitial score	0.33 ± 0.05	6.55 ± 0.81^**^	0 ± 0	3.12 ± 0.46^**#^
Glomerular collagen volume fraction (%)	0 ± 0	1.83 ± 0.45^**^	0 ± 0	1.15 ± 0.25^**#^
Renal tubular collagen volume fraction (%)	0 ± 0	0.138 ± 0.021^**^	0 ± 0	0.016 ± 0.002^**#^
BUN(mmol/L)	11.55 ± 1.17	30.20 ± 1.63^**^	10.77 ± 1.19	19.36 ± 1.98^**##^
Scr(umol/L)	7.12 ± 1.02	21.58 ± 2.29^**^	6.94 ± 0.82	15.78 ± 0.96^**##^
UPE (mg/d)	1.48 ± 0.12	10.73 ± 1.08^**^	1.52 ± 0.09	5.80 ± 1.04^**##^

*UPE* urinary protein excretion, *BUN* serum urea nitrogen, *Scr* serum creatinine

^**^P < 0.01*vs* WT-sham or C3^−/−^ sham, ^#^*P* < 0.05*vs* WT-AKI-CKD, ^#*#*^*P* < 0.01*vs* WT-AKI-CKD

Masson’s staining showed no blue stained tissues in the WT-sham group or the C3^−/−^-sham group. There were lots of blue staining in the WT-AKI-CKD group, whereas only a small amount of blue staining was observed in the C3^−/−^-AKI-CKD group (Fig. 2a). Compared with the WT-sham group, glomerular, renal tubular collagen volume fractions in the WT-AKI-CKD group were significantly increased (*P* < 0.01). There were substantial decline in the C3^−/−^-AKI-CKD group than in the WT-AKI-CKD group (*P* < 0.01) (Table 2).

Synaptopodin, Nephrin, and CD2AP immunohistochemical results showed a large number of brown-yellow granules in the glomeruli of the WT-sham group and C3^−/−^-sham group, while less in the WT-AKI-CKD group, and a moderate amount in the C3^−/−^-AKI-CKD group. The results of TRPC6 immunohistochemistry showed that only a small amount of brown-yellow granules was present in the glomeruli of the WT-sham group and the C3^−/−^-sham group, on the other hand, a large amount in the WT-AKI-CKD group and a moderate amount in the C3^−/−^-AKI-CKD group (Fig. 2b). Compared with the WT -AKI- CKD group, the IOD values of Synaptopodin, Nephrin, and CD2AP in the C3^−/−^-AKI- CKD group all increased significantly (*P* < 0. 01), while the TRPC6 IOD value decreased considerably (*P* < 0.01) (Fig. 2c).

Immunohistochemistry and Western blot analysis showed that, compared with the WT-sham group, the levels of Synaptopodin, Nephrin, and CD2AP in WT-AKI-CKD and C3^−/−^-AKI-CKD groups were obviously decreased (P < 0.01), while TRPC6 increased considerably (*P* < 0.01). Compared with WT -AKI- CKD group, the levels of Synaptopodin, Nephrin, and CD2AP were significantly increased (P < 0.01) and the level of TRPC6 protein considerably decreased in the C3^−/−^-AKI- CKD group (*P* < 0.01) (Fig. 2d, e).

TEM showed that a complete and clear podocyte structure with no foot process fusion was observed in the WT-sham and the C3^−/−^-sham group. There was extensive podocyte fusion, and podocyte segment dissection was observed in the WT-AKI-CKD group, whereas only a small amount of podocyte fusion was detected, and no podocyte segment dissection was seen in C3^−/−^-AKI-CKD group (Fig. 2f).

### C3 deficiency inhibited TLR4/NFκB-P65 signaling pathway activation post-IRI.

**Fig. 3:**
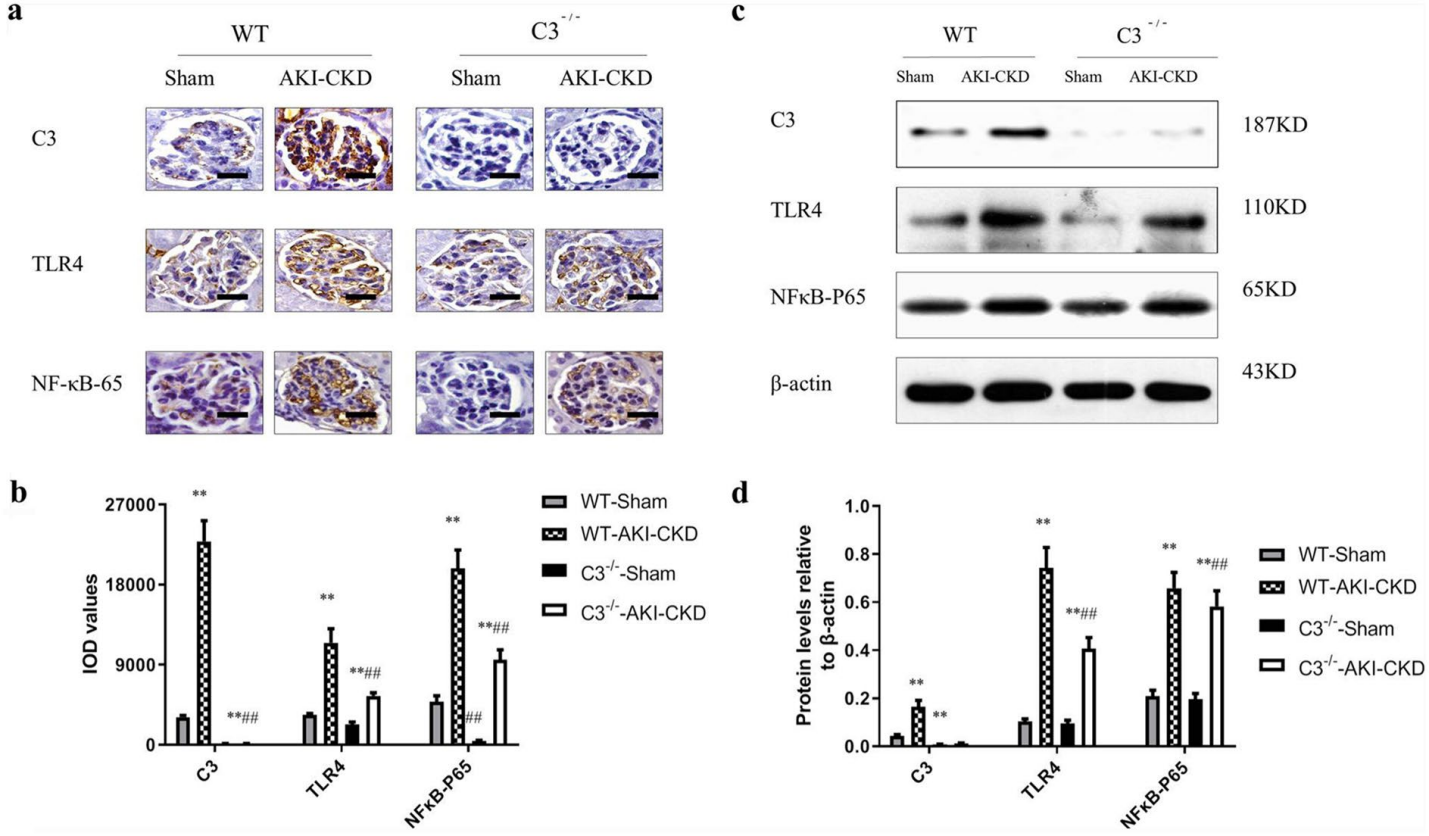
C3 deficiency inhibited TLR4 / NFκB-P65 signaling pathway activation during post-injury fibrosis. **a** Immunohistochemical detection of glomerular complement C3, TLR4, and NFκB-P65 protein expression (Scale bar = 25 μm) in each group of mice. **b** Renal tissue immunohistochemical staining optical density analysis, n = 10. **c** Western blot gel electrophoresis film exposure map, **d** Western blot gel electrophoresis exposure film grayscale analysis, with the target protein/β-actin (OD) ratio to express the target protein expression level, n = 10. **P < 0.01vs WT-sham or C3^−/−^-sham, ## *P* < 0.01vs WT-AKI -CKD

To verify the C3 promoting inflammation and therapeutic efficacy through the TLR4/NFκB-P65 pathway in vivo, we carried out quantitative and positional measurements. Immunohistochemistry and Western blot analysis showed that, compared with the WT-sham group, the levels of C3, TLR4, NFκB-P65 in the WT-AKI-CKD group were significantly increased (*P* < 0.01). Compared with the WT -AKI- CKD group, C3, TLR4, NFκB-P65 in the C3^−/−^-AKI-CKD group considerably decreased (*P* < 0.01) (Fig. 3a–d).

### The effect of C3a on podocyte cytoskeleton and signaling pathways under hypoxic-ischemic conditions

**Fig. 4:**
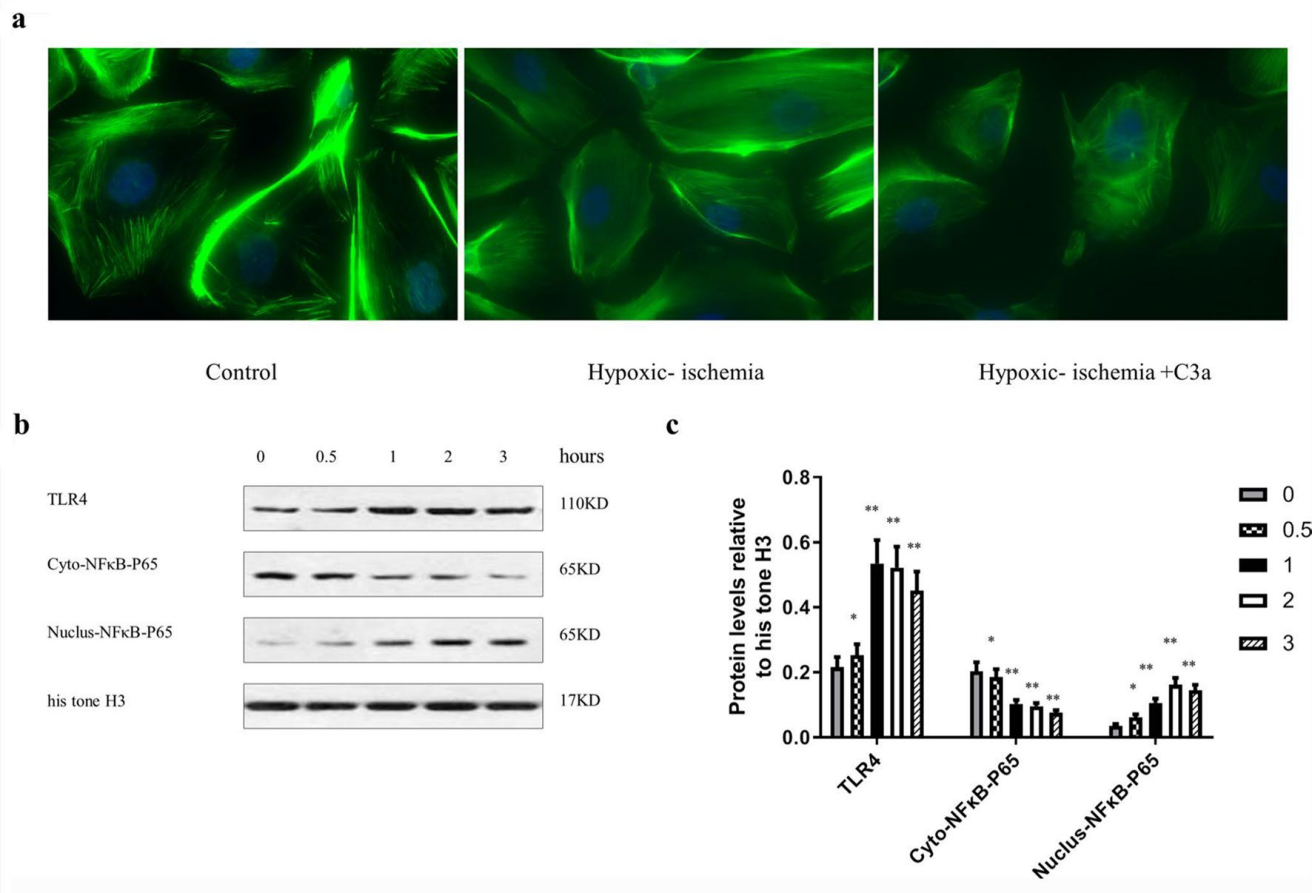
The effect of C3a on podocyte cytoskeleton and signaling pathways under hypoxic-ischemic conditions. **a** Podocyte cytoskeleton stained with phalloidin, objective lens (40 ×). **b** Immunoblotting film exposure map, **c** Immunoblotting exposure film optical density analysis, n = 5. *P < 0.05vs Control (C3a: 0 h) group. ** *P* < 0.01 vs Control (C3a: 0 h) group

To discern whether C3a promoted podocyte phenotypic alteration in hypoxic-ischemic, we exposed podocytes to C3a. Phalloidin staining showed that in the control group, cells were elongated or star shaped, had a small number of protrusions, and displayed regularly arranged cytoplasmic muscle filaments. Cell in the hypoxic-ischemic group demonstrated an irregularly shaped cell body, rounded cells, and disordered cytoplasmic muscle filament arrangement. In the hypoxic-ischemia + C3a group (C3a Merck Millipore, USA), the cell body was also irregularly shaped in a rounded manner and the arrangement of cytoplasmic muscle filaments was either disordered or unclear (Fig. 4a).

C3a promoted TLR4 expression and NFκB-P65 nuclear translocation in glomerular podocytes in a time-dependent manner under hypoxic-ischemia conditions. The level of TLR4 was significantly increased after 60 min with C3a (0.1 μM) under hypoxic-ischemia conditions (*P* < 0.01), and reaching its peak after 2 h. Meanwhile, cytoplasmic NFκB-P65 was significantly decreased (*P* < 0.01) and nucleus NFκB-P65 was markedly increased (Fig. 4b, c).

### Inhibition of TLR4 on C3a-induced podocyte damage and function under hypoxic-ischemic conditions

**Fig. 5:**
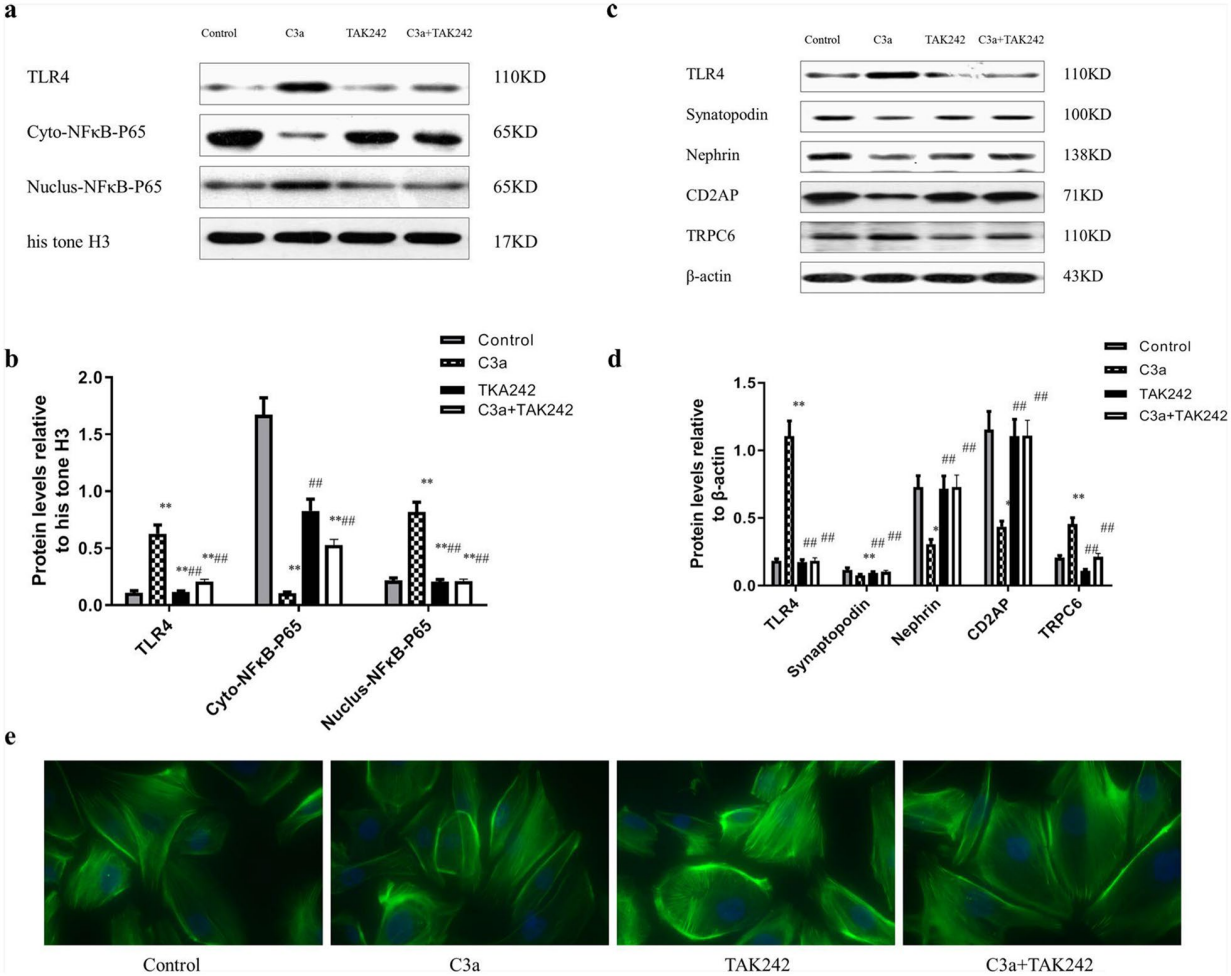
Inhibition of TLR4 on C3a-induced podocyte damage and function under hypoxic-ischemic conditions. **a** Immunoblotting film exposure map, **b** Immunoblotting exposure film optical density analysis, n = 3. **c** Immunoblotting film exposure map, **d** Immunoblotting exposure film optical density analysis. **e** Inhibition of the effect of TLR4 on the C3a-induced podocyte cytoskeleton under hypoxic-ischemic conditions: stained with phalloidin, objective lens (40 ×). ** P < 0.01 vs Control, ## *P* < 0.01 vs C3a (0.1 μM)

To determine whether TLR4-mediated C3a activation specifically contributed to exacerbated podocytes in hypoxic-ischemic, we used TAK242 to inhibit TLR4 expression. Compared with the Control group, the levels of TLR4 and nuclear NFκB-P65 in the C3a group were significantly increased (*P* < 0.01) and the TLR4 receptor inhibitor TAK242 (5 μM) inhibited TLR4 expression and NFκB-P65 nuclear translocation, and compared with the C3a group, TLR4 expression and nuclear NFκB-P65 levels of the C3a + TAK242 group were significantly decreased (*P* < 0.01) (Fig. 5a, b).

Compared with the control group, the levels of Synaptopodin, Nephrin, and CD2AP in the C3a group were markedly reduced (*P* < 0.01), but TLR4 and TRPC6 substantially increased (*P* < 0.01). Compared with the control group, the levels of TLR4, Synaptopodin, Nephrin, CD2AP, and TRPC6 in the TAK242 group were no statistical difference (*P* > 0.05). Compared with the C3a group, TLR4 and TRPC6 in the C3 + TAK242 group showed significantly reduced (*P* < 0.01), while Synaptopodin, Nephrin, and CD2AP were significantly elevated (*P* < 0.01) (Fig. 5c, d).

Phalloidin staining showed that in the control group, cells were elongated or star shaped with a few protrusions, and the cytoplasmic myofilaments were regularly arranged. In the C3a group, the cell body was irregular, with a rounded shape and disordered arrangement of cytoplasmic myofilament. In the TAK242 group, cells were elongated or star shaped, with a small number of protrusions and regularly arranged cytoplasmic muscle filaments. The cells were round or star shaped in the C3a+TAK242 group, with a regularly engineered cytoplasmic muscle filament (Fig. 5e).

### Effect of TLR4 up-regulation on C3a-induced podocyte function and hypoxic-ischemic damage.

**Fig. 6:**
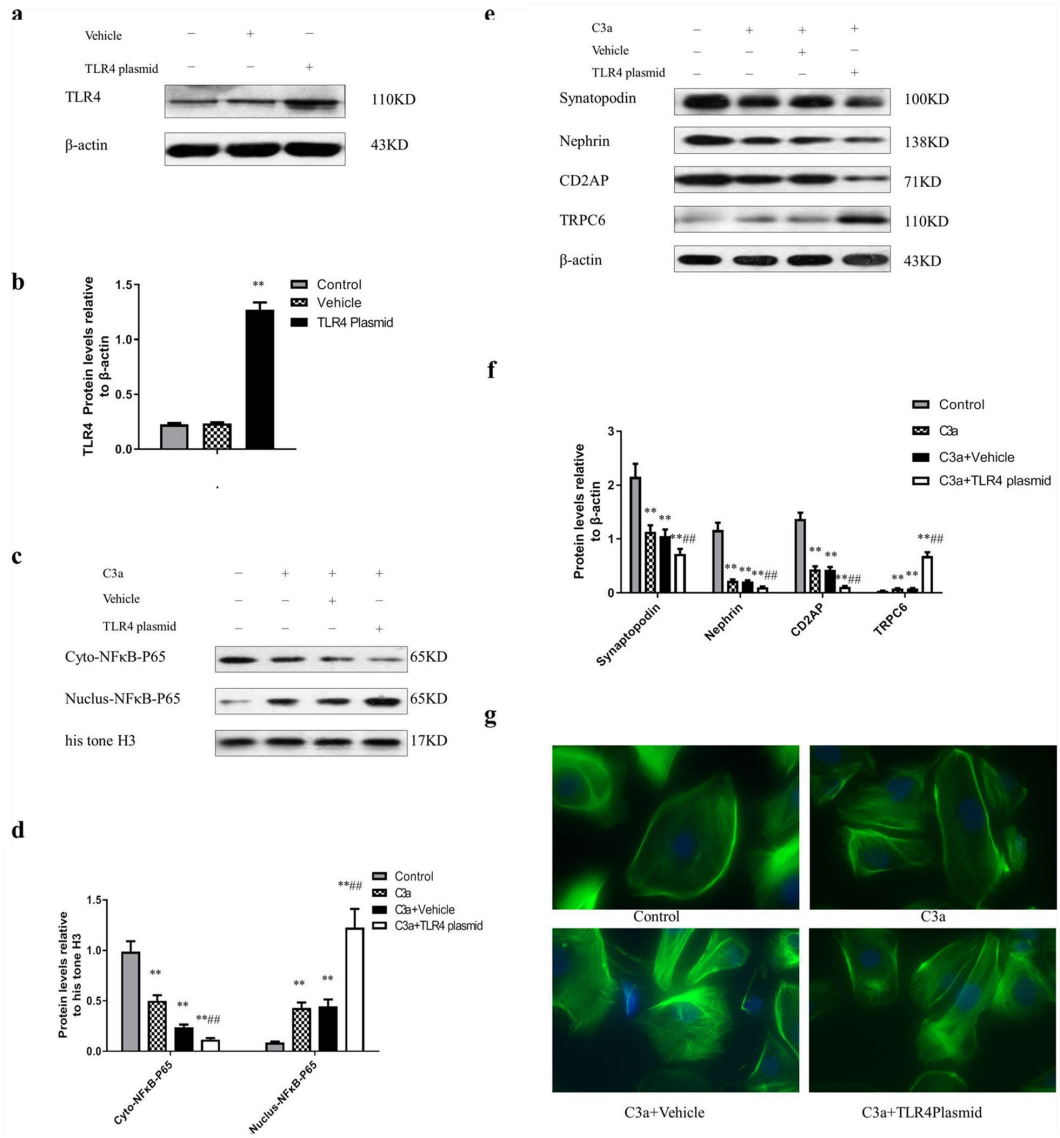
Effect of up-regulating TLR4 on C3a-induced podocyte damage and function under hypoxic-ischemic conditions. **a** Immunoblotting film exposure map; **b** Immunoblotting exposure film optical density analysis, n = 3. **c** Immunoblotting film exposure map; **d** Immunoblotting film optical density analysis, n = 3. **e** Immunoblotting film exposure image. **f** Immunoblotting exposure film optical density analysis, n = 4. **g** Podocyte cytoskeleton stained with phalloidin, objective lens (40 ×). ** *P* < 0.01 vs Control group; ## *P* < 0.01 vs C3a + Vehicle group

To further confirm the effect of TLR4 in hypoxic-ischemic, we overexpress the TLR4 in the podocyte. The TLR4 protein expression level was significantly increased in podocytes with TLR4 plasmid transfection (*P* < 0.01), and there was no statistically significant difference (*P* > 0.05) between empty plasmid group and liposome group and control group (Fig. 6a, b).

Compared with the vehicle group, the level of Cyto-NFκB-P65 protein in the C3a + TLR4 plasmid group (C3a 0.1 μM treatment) was significantly declined, the level of Nucleus-NFκB-P65 protein was considerably increased (*P* < 0.01) (Fig. 6c, d). The level of TRPC6 protein in the C3a+TLR4 plasmid group significantly decreased. On the other hand, Synaptopodin, Nephrin, and CD2AP protein expression levels were substantially increased (*P* < 0.01) (Fig. 6e, f).

Finally, phalloidin staining showed that in the Control group, cells were elongated or star shaped and had a small number of protrusions with regularly arranged cytoplasmic myofilaments. In the C3a group or C3a+Vehicle group, the cell body was irregularly shaped, with rounded cells and disordered or unclear cytoplasmic myofilaments. In the C3a+TLR4 plasmid group, the cell nucleus was markedly smaller, with both the membrane protrusion and the cytoplasmic myofilament disappearing (Fig. 6g).

## Discussion

At present, in the world, CKD's social and economic burden ranks among the top diseases [16, 17]. The prevalence of CKD in China has been increasing over the years [4, 17]. In recent years, epidemiological and experimental studies have shown that AKI is closely related to CKD, being one of the important factors attributed to the increased incidence of CKD [18–20]. This study found that complement C3 and the inflammation regulatory protein TLR4/NFκB-P65 all increased considerably after renal ischemia–reperfusion AKI and post-injury fibrosis. Complement C3 gene knockdown can mitigate the changes above. In vitro experiments showed that the inhibition of TLR4 can reduce C3a-induced podocyte NFκB-P65 levels under hypoxic-ischemic conditions, stabilizing the podocyte cytoskeleton. Moreover, up-regulating TLR4 levels have the opposite effect. We pointed out that complement C3a can mediate podocyte injury through TLR4/NFκB-P65 signaling pathway, participating in post-AKI fibrosis.

Recent study found that podocytes through the Wnt/β-Catenin pathway involve in AKI [8]. Our previous studies have found that podocyte damage may be the main cause of CKD after AKI [12]. Complement activation is involved in the pathogenesis of ischemia reperfusion injury (IRI), which is an inevitable process during AKI [21]. In investigating the causes of podocyte damage, we noticed that complement C3 is an important factor of damage. The Malmö Diet and Cancer cohort study found that in the general population, complement C3 was related to the incidence of first hospitalization of CKD [22]. The complement system activated, forming a membrane attack complex (MAC), resulting in podocyte apoptosis and damage, cytoskeletal changes, abnormal pore membrane protein, filtration barrier damage, and proteinuria [23, 24]. Recent studies have demonstrated that the pathogenic activation of complement by the glomerular subepithelial immune complex is a critical step for proteinuria. And the activation of complement is a key trigger for podocyte loss and activation of the mesangial epithelial cells of the glomeruli, which then lead to glomerulosclerosis [25, 26].

Infiltration of inflammatory cells and activation of inflammatory factors in kidney tissue after IRI have been shown to accelerate the progression of CKD. Studies by Chao Hu et al. [21] showed that complement activation induces renal IRI, and the complement inhibitor complement receptor of the immunoglobulin superfamily (CRIg) /FH can improve renal IRI by activating PI3K/AKT signaling. Targeting of a human complement inhibitor, CR1, provided effective protection against cardiac IRI [27]. Complements C3a and C5a could lead to endothelial cell transdifferentiation after IRI, and inhibition of complements C3a and C5a can significantly reduce renal fibrosis [28, 29]. C3a induces mitochondrial dysfunction in podocytes, and inhibition of C3aR significantly limited podocyte loss and enhanced podocyte density [30]. These data, together with the evidence that injury to podocytes is a major cause of glomerulosclerosis, support our speculation that there is a detrimental link between complement C3 and podocyte injury in the post-injury fibrosis of the kidney after AKI. Our study found that podocyte injury and extensive foot process fusion occurred in the WT-AKI-CKD group, with a decrease in the expression of the podocyte functional proteins and an increase in the expression of TRPC6, progressively increasing proteinuria and eventually chronic renal fibrosis and renal dysfunction. Compared with the WT-AKI-CKD group, in the C3^−/−^-AKI-CKD group, TEM shows improved podocyte fusion, with significantly increased the level of podocyte functional protein and decreased the level of TRPC6, consequently improving proteinuria and renal function. Pathology evaluation further indicated that mesangial hyperplasia, glomerular sclerosis, and renal tubule interstitial fibrosis declined, demonstrating significant improvement in kidney chronic fibrosis.

Primary cultured human podocytes and conditionally immortalized mouse podocytes can synthesize and secrete complements C1q, C1r, C2, C3, C7, complement factor H (CFH), CD59, C4bp, CD46, Protein S, complement receptor 2 (CR2), C1qR, C3aR, C5aR, and Crry under physiological conditions. The synthesis increases under the stimulation of inflammatory factor interferon γ, which is affected by various cytokines such as angiotensin II (Ang II), IL6, and TGF-β [31]. In this research, we found that complement C3 was activated during post-AKI renal fibrosis of mice as complement C3 knockout mice can improve renal tissue inflammation and podocyte damage during AKI, reducing post-AKI renal fibrosis. We speculate that the activation of complement C3 is thereby closely related to podocyte injury and post-AKI renal fibrosis.

TLR4 is an immunoinflammatory grade trigger protein involved in the signal transduction of the ganglion reaction [32, 33], and widely expressed on the surface of various immune cells, as well as in glomerular podocytes, mesangial cells, and renal tubular epithelial cells [34, 35]. TLR4 and the complement system are widely involved in the occurrence and progression of various diseases, such as renal IRI, AKI and diabetic nephropathy [36–38], which can activate the transcription and expression of NFκB-mediated complement factor, thereby initiating the inflammatory cascade [39, 40]. In vitro experiments, we have observed that under hypoxic-ischemic conditions the differentiation of glomerular podocytes can be induced. Complement C3a promotes TGF-β1 synthesis and the TLR4/ NFκB-P65 protein expression in glomerular podocytes. We also observed that C3a promotes TLR4 expression and NFκB-P65 nuclear translocation in glomerular podocytes under hypoxic-ischemic conditions. Up-regulating the level of TLR4 can increase the levels of NFκB-P65 in C3a-induced podocyte nucleus under hypoxic-ischemic conditions, inducing the synthesis of the glomerular podocyte transdifferentiation proteins Synaptopodin, Nephrin, and CD2AP and reducing the expression of TRPC6, resulting in obvious damage to the podocyte cytoskeleton. TLR4 inhibitors can inhibit NFKB-P65 nuclear translocation, reducing the protein expression level of Synaptopodin, Nephrin, and CD2AP, inducing TRPC6 protein synthesis, and stabilizing the podocyte cytoskeleton. Since podocytes express both the C3a receptor and TLR4, we speculate that complement C3a may activate the TLR4 protein of glomerular podocytes through the TLR4/NFκB-P65 signaling pathway, inducing NFκB-P65 nuclear translocation, participating in the inflammation and transdifferentiation of glomerular podocytes and resulting in the progression of post-AKI renal fibrosis.

## Conclusion

In summary, complement C3-mediated inflammatory response-induced glomerular podocyte injury is closely related to the post-injury fibrosis after renal ischemia–reperfusion AKI. These effects may be achieved by regulating podocyte TLR4/NFκB-P65 signaling pathway.

## Data Availability

The data used to support the findings of this study are available from the corresponding author upon reasonable request.

## Abbreviations

AKI: Acute kidney injury
Ang II: Angiotensin II
BUN: Blood urea nitrogen
CD2AP: CD2-associated protein
CFH: Complement factor H
CKD: Chronic kidney disease
CR2: Complement receptor 2
IRI: Ischemia–reperfusion injury
MAPKs: Activated protein kinases
RTIS: Renal tubulointerstitial score
Scr: Serum creatinine
TLR4: Toll-like receptor 4

## References

[1] GBD 2019 Diseases and Injuries Collaborators (2020). Global burden of 369 diseases and injuries in 204 countries and territories, 1990–2019: a systematic analysis for the Global Burden of Disease Study 2019. Lancet.

[2] Zhang L, Wang F, Wang L, Wang W, Liu B, Liu J, Chen M, He Q, Liao Y, Yu X, Chen N, Zhang JE, Hu Z, Liu F, Hong D, Ma L, Liu H, Zhou X, Chen J, Pan L, Chen W, Wang W, Li X, Wang H (2012). Prevalence of chronic kidney disease in China: a cross-sectional survey. Lancet.

[3] Fang Y, Teng J, Ding X (2015). Acute kidney injury in China. Hemodial Int.

[4] Zhang L, Zhao MH, Zuo L, Wang Y, Yu F, Zhang H, Wang H (2019). China Kidney Disease Network (CK-NET) 2015 Annual Data Report. Kidney Int Suppl.

[5] Zhou M, Wang H, Zeng X, Yin P, Zhu J, Chen W, Li X, Wang L, Wang L, Liu Y, Liu J, Zhang M, Qi J, Yu S, Afshin A, Gakidou E, Glenn S, Krish VS, Miller-Petrie MK, Mountjoy-Venning WC, Mullany EC, Redford SB, Liu H, Naghavi M, Hay SI, Wang L, Murray CJL, Liang X (2019). Mortality, morbidity, and risk factors in China and its provinces, 1990–2017: a systematic analysis for the Global Burden of Disease Study 2017. Lancet.

[6] Coca SG, Yusuf B, Shlipak MG, Garg AX, Parikh CR (2009). Long-term risk of mortality and other adverse outcomes after acute kidney injury: a systematic review and meta-analysis. Am J Kidney Dis.

[7] Horne KL, Packington R, Monaghan J, Reilly T, Selby NM (2017). Three-year outcomes after acute kidney injury: results of a prospective parallel group cohort study. BMJ Open.

[8] Hu X, Zhou W, Wu S, Wang R, Luan Z, Geng X, Xu N, Zhang Z, Ruan Z, Wang Z, Li F, Yu C, Ren H (2022). Tacrolimus alleviates LPS-induced AKI by inhibiting TLR4/MyD88/NF-κB signalling in mice. J Cell Mol Med.

[9] Markarian M, Krattli RP, Baddour JD, Alikhani L, Giedzinski E, Usmani MT, Agrawal A, Baulch JE, Tenner AJ, Acharya MM (2021). Glia-selective deletion of complement C1q prevents radiation-induced cognitive deficits and neuroinflammation. Cancer Res.

[10] Merle NS, Paule R, Leon J, Daugan M, Robe-Rybkine T, Poillerat V, Torset C, Frémeaux-Bacchi V, Dimitrov JD, Roumenina LT (2019). P-selectin drives complement attack on endothelium during intravascular hemolysis in TLR-4/heme-dependent manner. Proc Natl Acad Sci U S A.

[11] Wang K, Wei H, Zhan J, Liang X, Zhang C, Liu Y, Xu G (2020). GSPE alleviates renal fibrosis by inhibiting the activation of C3/ HMGB1/TGF-β1 pathway. Chem Biol Interact.

[12] Chen Y, Lin L, Tao X, Song Y, Cui J, Wan J (2019). The role of podocyte damage in the etiology of ischemia-reperfusion acute kidney injury and post-injury fibrosis. BMC Nephrol.

[13] Cui J, Wu X, Song Y, Chen Y, Wan J (2019). Complement C3 exacerbates renal interstitial fibrosis by facilitating the M1 macrophage phenotype in a mouse model of unilateral ureteral obstruction. Am J Physiol Renal Physiol.

[14] Shankland SJ, Pippin JW, Reiser J, Mundel P (2007). Podocytes in culture: past, present, and future. Kidney int.

[15] Sauvant C, Schneider R, Holzinger H, Renker S, Wanner C, Gekle M (2009). Implementation of an in vitro model system for investigation of reperfusion damage after renal ischemia. Cell Physiol Biochem.

[16] Webster AC, Nagler EV, Morton RL, Masson P (2017). Chronic Kidney Disease. Lancet.

[17] Bowe B, Xie Y, Li T, Mokdad AH, Xian H, Yan Y, Maddukuri G, Al-Aly Z (2018). Changes in the US Burden of Chronic Kidney Disease From 2002 to 2016: An Analysis of the Global Burden of Disease Study. JAMA Netw Open.

[18] Hapca S, Siddiqui MK, Kwan RSY, Lim M, Matthew S, Doney ASF, Pearson ER, Palmer CNA, Bell S (2021). The Relationship between AKI and CKD in Patients with Type 2 Diabetes: An Observational Cohort Study. J Am Soc Nephrol.

[19] Chawla LS, Eggers PW, Star RA, Kimmel PL (2014). Acute kidney injury and chronic kidney disease as interconnected syndromes. N Engl J Med.

[20] Venkatachalam MA, Weinberg JM, Kriz W, Bidani AK (2015). Failed Tubule Recovery, AKI-CKD Transition, and Kidney Disease Progression. J Am Soc Nephrol.

[21] Hu C, Li L, Ding P, Li L, Ge X, Zheng L, Wang X, Wang J, Zhang W, Wang N, Gu H, Zhong F, Xu M, Rong R, Zhu T, Hu W (2018). Complement Inhibitor CRIg/FH Ameliorates Renal Ischemia Reperfusion Injury via Activation of PI3K/AKT Signaling. J Immunol.

[22] Bao X, Borné Y, Muhammad IF, Schulz CA, Persson M, Orho-Melander M, Niu K, Christensson A, Engström G (2019). Complement C3 and incident hospitalization due to chronic kidney disease: a population-based cohort study. BMC Nephrol.

[23] Danobeitia JS, Djamali A, Fernandez LA (2014). The role of complement in the pathogenesis of renal ischemia-reperfusion injury and fibrosis. Fibrogenesis Tissue Repair.

[24] Yu SM, Nissaisorakarn P, Husain I, Jim B (2018). Proteinuric Kidney Diseases: A Podocyte's Slit Diaphragm and Cytoskeleton Approach. Front Med (Lausanne).

[25] Morigi M, Locatelli M, Rota C, Buelli S, Corna D, Rizzo P, Abbate M, Conti D, Perico L, Longaretti L, Benigni A, Zoja C, Remuzzi G (2016). A previously unrecognized role of C3a in proteinuric progressive nephropathy. Sci Rep.

[26] Luo W, Olaru F, Miner JH, Beck LH, van der Vlag J, Thurman JM, Borza DB (2018). Alternative Pathway Is Essential for Glomerular Complement Activation and Proteinuria in a Mouse Model of Membranous Nephropathy. Front Immunol.

[27] Zheng C, Sleiman MM, Yang X, He S, Atkinson C, Tomlinson S (2021). Increasing the efficacy and safety of a human complement inhibitor for treating post-transplant cardiac ischemia reperfusion injury by targeting to a graft-specific neoepitope. J Heart Lung Transplant.

[28] Curci C, Castellano G, Stasi A, Divella C, Loverre A, Gigante M, Simone S, Cariello M, Montinaro V, Lucarelli G, Ditonno P, Battaglia M, Crovace A, Staffieri F, Oortwijn B, van Amersfoort E, Gesualdo L, Grandaliano G (2014). Endothelial-to-mesenchymal transition and renal fibrosis in ischaemia/reperfusion injury are mediated by complement anaphylatoxins and Akt pathway. Nephrol Dial Transplant.

[29] Durigutto P, Sblattero D, Biffi S, De Maso L, Garrovo C, Baj G, Colombo F, Fischetti F, Di Naro AF, Tedesco F, Macor P (2017). Targeted Delivery of Neutralizing Anti-C5 Antibody to Renal Endothelium Prevents Complement-Dependent Tissue Damage. Front Immunol.

[30] Morigi M, Perico L, Corna D, Locatelli M, Cassis P, Carminati CE, Bolognini S, Zoja C, Remuzzi G, Benigni A, Buelli S (2020). C3a receptor blockade protects podocytes from injury in diabetic nephropathy. JCI Insight.

[31] Li X, Ding F, Zhang X, Li B, Ding J (2016). The Expression Profile of Complement Components in Podocytes. Int J Mol Sci.

[32] Molteni M, Gemma S, Rossetti C (2016). The Role of Toll-Like Receptor 4 in Infectious and Noninfectious Inflammation. Mediators Inflamm.

[33] Yang Y, Lv J, Jiang S, Ma Z, Wang D, Hu W, Deng C, Fan C, Di S, Sun Y, Yi W (2016). The emerging role of Toll-like receptor 4 in myocardial inflammation. Cell Death Dis.

[34] Mudaliar H, Pollock C, Panchapakesan U (2014). Role of Toll-like receptors in diabetic nephropathy. Clin Sci (Lond).

[35] McCoy KL (2016). Interaction between Cannabinoid System and Toll-Like Receptors Controls Inflammation. Mediators of inflammation. Mediators Inflamm.

[36] Gluba A, Banach M, Hannam S, Mikhailidis DP, Sakowicz A, Rysz J (2010). The role of Toll-like receptors in renal diseases. Nat Rev Nephrol.

[37] Damman J, Daha MR, van Son WJ, Leuvenink HG, Ploeg RJ, Seelen MA (2011). Crosstalk between complement and Toll-like receptor activation in relation to donor brain death and renal ischemia-reperfusion injury. Am J Transplant.

[38] Zhao H, Perez JS, Lu K, George AJ, Ma D (2014). Role of Toll-like receptor-4 in renal graft ischemia-reperfusion injury. Am J Physiol Renal Physiol.

[39] Rao J, Yue S, Fu Y, Zhu J, Wang X, Busuttil RW, Kupiec-Weglinski JW, Lu L, Zhai Y (2014). ATF6 mediates a pro-inflammatory synergy between ER stress and TLR activation in the pathogenesis of liver ischemia-reperfusion injury. Am J Transplant.

[40] Zuniga MC, Raghuraman G, Hitchner E, Weyand C, Robinson W, Zhou W (2017). PKC-epsilon and TLR4 synergistically regulate resistin-mediated inflammation in human macrophages. Atherosclerosis.

